# The Small Non-Coding RNA Profile of Human and Mouse Sperm

**DOI:** 10.3390/ncrna11010015

**Published:** 2025-02-09

**Authors:** Yoon Sing Yap, Pasquale Patrizio, Luisa Cimmino, Konstantinos Sdrimas, Aristeidis G. Telonis

**Affiliations:** 1 Department of Biochemistry and Molecular Biology, University of Miami Miller School of Medicine, Miami, FL 33136, USA; yxy957@med.miami.edu (Y.S.Y.); luisa.cimmino@med.miami.edu (L.C.); 2 Sylvester Comprehensive Cancer Center, University of Miami Miller School of Medicine, Miami, FL 33136, USA; 3 Department of Obstetrics, Gynecology, Reproductive Sciences, University of Miami Miller School of Medicine, Miami, FL 33136, USA; pxp612@med.miami.edu; 4 Department of Medical Oncology, West Virginia Cancer Institute, West Virginia University, Morgantown, WV 26506, USA; konstantinos.sdrimas1@hsc.wvu.edu

**Keywords:** sperm, tRNA fragments, rRNA fragments, miRNA, epigenetics

## Abstract

Small non-coding RNAs constitute a dynamic epigenetic layer in mature spermatozoa that can exert transgenerational regulatory functions. Here, we review recent advances in the field of small RNAs in spermatozoa, how their profiles change in response to lifestyle or environmental factors, and their impact on offsprings’ physiology. The profile of these RNAs changes dramatically during spermatozoa maturation. The majority of intracellular small RNAs during early spermatogenesis are miRNAs and piRNAs, but, in mature spermatozoa, tRNA- and rRNA-derived fragments (tRFs and rRFs, respectively) are the predominant forms, primarily delivered from the epididymis via extracellular vesicles. Diet, exercise, and environmental exposures have a direct effect on small RNA levels in spermatozoa, and this differential abundance can reprogram the development of the embryo. Offsprings of fathers with different lifestyles can have different phenotypes, including altered metabolism or behavior. Therefore, small RNAs in spermatozoa are emerging as an important epigenetic layer in development and transgenerational inheritance.

## 1. Introduction

The cell’s transcriptome constitutes a complex biological layer with tens of thousands of RNAs, including both coding and non-coding RNAs. Specifically, the most recent assembly of the human genome includes 19,868 coding genes, 42,160 non-coding genes, and 15,206 pseudogenes (Ensembl release 113) [1]. Thus, at least two non-coding genes account for every coding gene. Transcriptomic complexity further increases when we consider alternative transcript variants: 387,944 distinct transcripts are listed for the human genome, an order of magnitude greater than the number of genes. Even when focusing on small RNAs—by convention, any RNA molecule less than 200 base pairs—there is still great heterogeneity and complexity. This group of molecules includes micro RNAs (miRNAs) [2,3], transfer RNAs (tRNAs) [4], and ribosomal RNAs (rRNAs) [5].

The mechanisms of the transcription, maturation, and processing of coding genes and transcripts seem unpretentious when compared to the variety in the biogenesis pathways and functions of small non-coding RNAs (sncRNAs). Each family of sncRNAs exhibits distinct origin dynamics and dependencies. Derived from RNA polymerase II, miRNAs have well-documented roles in regulating the abundance of messenger RNAs (mRNAs) or other long RNAs through RNA interference (RNAi) mechanisms. Transcribed from RNA polymerases I and III, rRNAs and tRNAs are central components in the expression of genetic information, but these molecules are not to be merely considered as housekeeping molecules. There are different types of tRNAs, even for the same amino acid [6], and different types of ribosomal RNA/protein complexes. The pools of tRNAs and of ribosomes are dynamic and responsive to external signals, stress, or differentiation [7,8,9,10]. Furthermore, one miRNA, tRNA, or rRNA gene does not only produce one transcript/product, even if the gene contains no introns. These molecules can be cleaved and produce a series of different isoforms or tRNA- and rRNA-derived fragments (tRFs and rRFs, respectively) with distinct molecular functions depending on the biological context [11,12,13,14].

Germ cells are unique cells in the sense that they are destined to give rise to the offspring and transmit genetic information to the next generation. It is without surprise that the functions of sncRNAs in germ cells, and specifically in spermatozoa, are considerably different than in somatic cells. We aim to review if even subtle deviations in the levels of sncRNA in spermatozoa can have long-lasting effects and affect the physiology of the offspring.

Spermatogenesis is a highly orchestrated biological process during which spermatogonia stem cells differentiate and go through meiosis to form mature spermatozoa. This process involves the packaging of DNA around protamines and the depletion of large parts of the cytoplasm, including ribosomal RNAs and organelles [15,16]. As spermatogenesis progresses, certain gene groups significantly change in expression. There is a transient upregulation of ribosomal and meiotic genes, followed by the overexpression of spermatogenesis-specific genes, like *PRM3*, while ribosomal genes are downregulated overall [17]. Although protein synthesis is depleted in mature spermatozoa, mammalian sperm contains intact coding transcripts [18]. Additionally, hundreds of small and long non-coding RNAs have been characterized, mostly of unknown function [18,19].

Here, our focus will be on the small RNA repertoire found in mature spermatozoa. We searched PubMed and GEO databases for studies that describe the small non-coding “RNAome”, i.e., the totality of sncRNAs as captured by the current extraction protocols, library construction methodologies, sequencing platforms, and bioinformatic analyses. We specifically focus on studies that include analyses not restricted to one type of molecules, e.g., miRNAs, but ones that are able to provide a holistic perspective on sperm sncRNA profiles. First, we review recent scientific findings on the content of small RNAs in human or other mammals’ spermatozoa. Then, we present the known evidence on the biogenesis and origin of the major classes of small RNAs in sperm and how lifestyle or environmental perturbation may lead to different compositions. Finally, we discuss how these molecules may affect the phenotype of the offspring and be the basis of epigenetic transgenerational inheritance.

## 2. The Small RNAome in Spermatozoa

The content of sncRNAs in mammalian spermatozoa has been the subject of multiple recent studies [20,21,22,23,24,25,26]. There is overall agreement that tRFs and rRFs are the most prevalent, although the exact percentages differ across studies, while miRNAs constitute a small fraction of all sncRNAs (Table 1). Analyzing spermatozoa from individuals of Han Chinese nationality aged 24–50 years old, Hua et al. found that almost half of the spermatozoa sncRNA are tRFs, mostly from the 5′ endpoint of the mature tRNA molecule (5′-tRFs and 5′-tRNA halves) from glycine (Gly), glutamate (Glu), and lysine (Lys) tRNAs [22]. Natt et al. collected samples at the University of Linköping in Sweden, from men aged 20–30 years old, and reported that almost 75% of the sncRNA spermatozoa content is rRFs, while tRFs constitute about 10% of all sncRNAs (5′-tRFs from Glu, Gly, valine, Val, and tRNAs) and around 6% are miRNAs [23]. Further, the study of Hamilton et al. on normozoospermic individuals aged 22–64 years [21] found that more than half of the sncRNA in spermatozoa is rRFs, while tRFs constitute an average of 18% and miRNAs 7% of the total profile.

The variability in the type of sncRNAs observed in human spermatozoa also exists in rodent studies. Sharma et al. used FVB/NJ mice and quantified their sncRNA profile [24]. They reported that tRFs constitute the biggest group, accounting for almost 80% of the molecules in mouse spermatozoa; most of these tRFs are 5′-tRFs and 5′-tRNA halves from tRNAs with anticodons of Glu and Gly. Similarly, in C57BL/6N mice, Tomar et al. found that about half of all spermatozoa sncRNAs are tRFs, while each of the rRFs and miRNAs constitute about 10% [27]. However, C57BL/6 mice spermatozoa content analyzed by Short et al. [25] was reported to be primarily constituted by miRNAs (50%) followed by tRFs (~15%) and by rRFs (~10%). A most recent study by Shaffer et al. in mice reported that the majority (almost 80%) of sncRNAs are derived from rRNAs, while tRFs constitute about 10% of all sncRNAs [28]. In Wistar rats, Suvorov et al. found that 40% of the rat’s small RNAs in spermatozoa are rRFs, and ~15% originate from tRNAs, while miRNAs make up 5–10% of all sncRNAs [26].

The above studies in humans and rodents highlight the significant variation in assessing spermatozoa sncRNAs. Although technical aspects, like library and sequencing protocols or batch effects, cannot be excluded, a series of lifestyle factors may significantly affect the biogenesis and relative levels of sncRNAs. An additional parameter to consider are ethnic/racial and geographical variations that may contribute to the observed heterogeneity. Given the considerably dynamic nature of spermatozoa, further large-scale time-series studies would be needed to widely map the sncRNA landscape in these cells as they complete spermatogenesis and their epididymal transit.

**Table 1:** Overview of studies on human and rodent sncRNAs in sperm.

Study	Organism	Population/Strain	Type of Sperm	RNA Extraction Method	Library Preparation Method	Top sncRNA Categories
Hua et al. [22]	Human	Han Chinese nationality; 24–50 years old	motile spermatozoa	Trizol	Illumina’s small RNA	tRFs (56%); rRFs (18%); miRNAs (6%)
Natt et al. [23]	Human	University of Linköping, Sweden; 20–30 years old	motile spermatozoa	miRNeasy Micro kit (Qiagen, Hilden, Germany)	NEBNext small RNA library Prep Set for Illumina	rRFs (73%); tRFs (10%); miRNAs (6%)
Hamilton et al. [21]	Human	CReATe Fertility Centre, Canada; 22–64 years old	fresh ejaculate	Rneasy Kit (Qiagen)	NEXTFLEX Small RNA-Seq Kit v3	rRFs (55%); tRFs (18%); miRNAs (7%)
Sharma et al. [24]	Mouse	FVB/NJ	cauda spermatozoa	TRI reagent	Small RNA cloning	tRFs (~80%); miRNAs (~10%); piRNAs (~10%)
Shaffer et al. [28].	Mouse	FVB/NJ	cauda spermatozoa	Trizol	PNK treatment and OTTR-seq	rRFs (80%); tRFs (10%); miRNAs (10%)
Tomar et al. [27]	Mouse	FVB/NJ	cauda spermatozoa	RNAeasy Mini Kit (Qiagen)	NEBNext Multiplex Small RNA Library Prep Set for Illumina	tRFs (~50%); rRFs (~10%); miRNAs (~10%)
Short et al. [25]	Mouse	C47BL/6	motile spermatozoa	QIAzol lysis reagent (Qiagen)	Illumina’s protocols	miRNAs (50%); tRFs (~15%); rRFs(~10%)
Suvorov et al. [26]	Rat	Wistar rats	motile spermatozoa	RNeasy Mini Kit (Qiagen)	NEBNext Multiplex Small RNA Library Prep Set for Illumina	rRFs (40%); tRFs (15%); miRNAs (5%)

## 3. Biogenesis and Origin of Small RNAs in Spermatozoa

**Figure 1:**
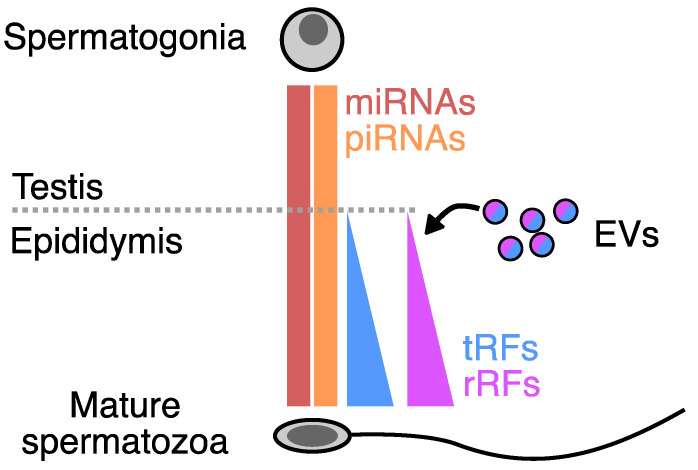
Overview of relative levels of small non-coding RNA as spermatogonia differentiate to mature spermatozoa based on current literature findings. As they pass through the epididymis, spermatozoa are loaded with small non-coding RNAs from extracellular vesicles derived from epididymal cells.

The profiling of the sncRNA content in mature spermatozoa provides a snapshot of the final output of all biogenetic mechanisms at play during spermatogenesis. The sncRNA profile in mature spermatozoa is not merely residual RNA from spermatogonia, but its content specifically changes as spermatogenesis progresses and even when spermatozoa pass through the epididymis (Figure 1).

Among the pioneering studies in the field was that of Sharma et al., where it was reported that sncRNA profiles are significantly different depending on the tissue from which sperm is isolated [24]. Specifically, testis spermatozoa primarily contain piRNAs, while cauda epididymal spermatozoa is enriched in tRFs. These tRFs are delivered from epididymal cells through extracellular vesicles (EVs). Subsequent studies further elucidated the biogenetic mechanisms and shed light on how epididymosomes deliver tRFs to the maturing sperm while miRNAs originate intracellularly [29].

In this context, further studies showed that altering conditions that affect epididymal cells is sufficient in changing the content of EVs and, in turn, of spermatozoa sncRNAs. Chan et al. demonstrated that the content of EVs from epididymal cells can be altered by corticosterone treatment and that those EVs are delivered to maturing spermatozoa [20]. Tomar et al. tracked the profiles of sncRNAs in spermatozoa along the testis–epididymal track and correlated which of these profiles respond to high-fat diet [27]. Their findings on the changes in spermatozoa sncRNAs, particularly of tRFs, in the epididymis further argue on the important role of epididymal-derived sncRNAs in shaping the RNA profiles of mature spermatozoa. Finally, Shaffer et al. knocked-out (KO) the enzymes that are believed to be responsible for the biogenesis of tRFs, specifically RNases 9, 10, 11, and 12 [28], resulting in a global downregulation of certain classes of sncRNAs, particularly of tRFs in mature mouse spermatozoa. These tRFs originated from several different parental tRNAs and were from different classes. By contrast, the levels of miRNAs did not significantly change. A considerable portion of these tRFs was also differentially expressed in the epididymis after RNase KO, suggesting the uptake of these molecules from epididymal cells.

Collectively, as spermatozoa transit from the testis through the epididymis, it is loaded with certain sncRNAs, particularly tRFs. This loading seems to occur through EVs from epithelial epididymal cells, as genetic or environmental perturbations have a direct effect on these cells and the levels of tRFs they generate.

## 4. Lifestyle and Environmental Effects on RNA in Spermatozoa

The abundance and relative composition of spermatozoa in RNAs change as a response to a series of environmental factors, including diet, aging, or stress, highlighting the dynamic nature of spermatozoa sncRNA.

The effect of diet and nutrition have arguably gathered a lot of research attention. Sharma et al. examined the effect of low-protein diet in male mice and found that it results in the upregulation of 5′-tRFs from Glu, Gly, Lys, and histidine (His) tRNAs in spermatozoa, while miRNAs, like let-7, are downregulated [24]. Tomar et al. focused on the effect of high-fat diet on mouse spermatozoa versus mice fed a low-fat diet. They documented 25% of sncRNAs change in response to diet [27]. The effect was more prominent in tRFs and depended on the organelle from which they originated: mitochondrially derived tRFs were upregulated, while nuclear-encoded tRFs were downregulated.

Human spermatozoa sncRNAs also respond to diet. In a paired analysis of the same donors, Natt et al. found that a one-week sugar-rich diet led to the increase in tRFs, primarily i-tRFs and 3′-tRFs from eight isodecoders, including nuclear Lys, His, and threonine (Thr) and mitochondrial His, Thr, and serine (Ser) tRNAs, as well as miRNAs, while rRFs were found decreased [23]. The authors were able to correlate spermatozoa motility parameters with the levels of sncRNAs, arguing for the importance of examining molecular markers even when slight changes in clinical/routine parameters are observed.

Aging has a drastic effect in multiple layers of the body’s physiology, including spermatozoa motility. Thus, it has been reasonable to hypothesize that the spermatozoa sncRNA profiles would also differ in aged compared to young individuals. Using rat models and comparing postnatal day 65 to day 120, Suvorov et al. reported on the effect of aging in mature spermatozoa and found that the levels of rRFs declined with aging but tRFs exhibited an overall increase, including tRFs from tRNAs decoding Arg, Leu and Ser amino acids; piRNAs did not show significant trends with aging [26]. In aging mouse spermatozoa, Chan et al. found a wide range of changes in the miRNAs at 20 weeks old compared to 9-week-old mice [20], while Guo et al. reported age-dependent changes (14–18 months old compared to 3–4-month-old mice) in the expression of tRFs in mature spermatozoa, mostly 5′-tRFs from a series of tRNAs, including Ser, Gly, Glu, and His [30].

Chan et al. further examined how chronic stress, a 4-week exposure in a random combination of adverse environments, including 36 h constant lighting and 1 h exposure to predator odor, affects the spermatozoa sncRNA profiles [20]. These stressors caused the reprogramming of sncRNA profiles in the short term but particularly in the long term, i.e., in the spermatozoa of aged individuals. In the context of environmental stress, McSwiggin et al. injected F0 female rats with toxicants and tracked the changes in the spermatozoa of F3 generation [31]. They found that hundreds to thousands of sncRNAs changed in response to toxicant injections, including tRFs, rRFs, and miRNAs, demonstrating the transgenerational inheritance of environmental exposures.

These studies highlight the importance of the environment in shaping the sncRNA profiles of the male gametes. The epididymis emerges as a sensitive sensor of environmental exposures and lifestyle that responds by loading spermatozoa with specific sncRNAs, particularly tRFs.

## 5. Effect on the Offsprings

The presence of sncRNAs in spermatozoa and their dynamic nature have sparked interesting hypotheses as key players for the transgenerational inheritance of environmental or lifestyle factors (Table 2). Indeed, sncRNAs are necessary for the fertility and proper development of the embryo [32,33,34], and, since they reprogram development without altering DNA sequence, they are considered epigenetic factors.

Proving transgenerational epigenetic inheritance requires carefully designed experiments [35,36,37]. The use of human cohorts is inherently biased due to cultural and socioeconomic backgrounds. In addition, sncRNAs that are identified as carriers of epigenetic inheritance are also necessary for sterility and embryo viability; thus, distilling the epigenetic effect on transgenerational inheritance becomes complicated. However, animal models with controlled environmental exposures provided significant insight on the epigenetic effect of sncRNAs.

Short et al. reported that male mice that exercised produced male offspring with reduced fear and anxiety, potentially through the upregulation of 5′-tRFs and 5′-tRNA halves from Gly tRNAs in the sperm [25]. To directly assess the effect of sncRNAs, Guo et al. injected small RNAs from aged and young mice into the zygote; the majority of the differentially expressed molecules between the two age groups were 5′-tRFs. Aged-derived spermatozoa resulted in higher anxiety markers in F1 male mice, as well as almost 500 differentially expressed genes in their cortex and hippocampus, particularly signaling and developmental genes. In an effort to pinpoint the origin of the transgenerational effect of sncRNAs, Chan et al. treated spermatozoa with EVs from epidydimal epithelial cells exposed to corticosterone, which alters their sncRNA content [20]. They used these spermatozoa for ICSI (intracytoplasmic sperm injection) to fertilize eggs and track the gene expression patterns of mid-gestation brain of the offspring. They found that the spermatozoa incubated with EVs from corticosterone-treated cells produced offspring that had higher expressions of synaptic signaling, neurotransmitter transport, and neurodevelopment and lower expressions of interferon and immune response genes. At puberty, the F1 generation had increased body weight and increased stress responses as adults.

In addition to long-term physiological effects, there have been documented molecular consequences in embryos stemming from the differential loading of spermatozoa sncRNAs. The study of Guo et al. also examined the expression profiles of pre-implantation embryos after the injection of small RNAs from aged mice and found thousands of differentially expressed genes, including genes in the MAPK, Ras, mTOR, and PI3K-Akt pathways [30]. The study of Tomar et al. traced that spermatozoa from high-fat diet fathers caused the upregulation of oxidative phosphorylation genes in mouse embryos [27], while Sharma et al. reported that the upregulation of 5′-tRFs, including from Glu and Gly tRNAs, and the downregulation of let-7 species in spermatozoa causes the repression of mouse endogenous retroviral elements (MERVLs) in the zygote [24], suggesting critical functions of these tRFs in core embryonic processes where MERVL are involved, like zygotic genome activation [38] or the epigenetic silencing of retrotransposons [39].

Overall, recent studies have shed light on how spermatozoa sncRNAs play a critical role in embryo viability and embryo quality [34]. Spermatozoa lacking sncRNAs are often considered as causes of infertility, while different levels of sncRNAs, including tRFs, rRFs, and miRNAs, lead to different levels of embryo quality as measured in in vitro fertilization (IVF) treatments [22,33].

## 6. Circular RNAs Emerge as Potent Conveyors of Transgenerational Epigenetic Inheritance

So far, we focused on non-coding RNAs with relatively small size, typically less than 100 nucleotides. However, circular RNAs (circRNAs) emerge as important factors in transgenerational epigenetic inheritance. CircRNAs are produced from backsplicing, a process where the 5′ and 3′ endpoints of different exons are excised from the pre-mRNA and ligated to form a circular RNA molecule. Their functions are multilevel, including sponging sncRNAs or RNA binding proteins [40,41,42,43,44]. As circRNAs levels in mature spermatozoa are also affected by environmental exposures, their mention is warranted in this review.

The biogenesis of circRNAs and their deposition in mature spermatozoa is not due to random errors of the splicing machinery. For instance, Chioccarelli et al. studied the biogenesis of one specific circRNA, circCNOT6L, and identified the RNA binding protein FUS as a necessary protein for the backsplicing of the *Cnot6L* mRNA [45]. Although not translated, this circRNA was shown to be transmitted to the zygote, with potential roles in zygotic genome activation.

Furthermore, environmental factors can have a significant effect on the levels of circRNAs in mature spermatozoa. Mele et al. investigated sperm quality parameters and circRNA levels in men exposed to toxic environmental contaminants and found that the levels of circRNAs in spermatozoa significantly correlated with the exposure levels to heavy metals [46]. The levels of circRNAs are also dynamic during aging, as thousands of circRNAs are differentially expressed in spermatozoa between young and aged individuals [47].

In summary, there is a growing body of evidence on the physiological and dynamic nature of circRNAs in spermatogenesis. Given their potentially extensive cross-talk with sncRNAs, a deeper understanding of circRNAs has the potential to fill gaps in the function of non-coding RNAs and their involvement in transgenerational epigenetic inheritance.

## 7. Conclusions and Open Questions

The technological advancements of high-throughput sequencing have propelled research output in the context of transgenerational inheritance of environmental and lifestyle factors. The sncRNA profiles in spermatozoa are highly dynamic and responsive to external factors, including diet, exercise, or stress, and we have only started to understand how they contribute to epigenetic transgenerational inheritance and in shaping the physiology of the offspring.

The sncRNA profile is considerably complex and heterogeneous. A multitude of molecules are not only produced by spermatogonia but are also delivered through EVs from supporting cells and tissues. The published research so far hints towards multiple dependencies of the sncRNA sperm profile, spanning from race and ethnicity to diet and exercise. Thus, interventions can cause rapid responses, whereby even one week of sugar-rich diet significantly rewires the sncRNAs. But the responses can be long-lasting as chronic stress seems to leave a permanent signature on the expression levels of spermatozoa sncRNAs.

The effect of differential levels of spermatozoa sncRNAs on the embryo molecular biology seems to be drastic and, depending on the context, seems to leave a permanent mark on the physiology of the offspring. However, the exact molecular mechanisms remain elusive. The current evidence suggests that sncRNAs, particularly tRFs, target ERV elements and/or other categories of repeat elements, like Alu elements. The expression of tRFs is strongly correlated with ERVs or Alu elements in somatic tissues and the genes that are rich in these elements [48,49,50], while zygotic genome activation and stemness genes are associated with high expression of retrotransposons and retrotransposon-dense genes [38,51]. Thus, the current data make it intriguing to build hypotheses on how transgenerational inheritance works on an epididymis–sperm–zygotic stemness regulatory axis.

We should note, here, that spermatozoa sncRNAs are not only a result of the recent environment and lifestyle of the individual. It has become apparent in mice that siblings have more similar sncRNA profiles than non-siblings exposed to the same diet [24]. However, this observation may not necessarily be explained solely by the common genetic background. A common nurturing environment may also be responsible. Nevertheless, the underlying, currently unknown complex interactions of the genome, the epigenome, and the environment in shaping the spermatozoa sncRNA profile and, by extension, the phenotype constitute an exciting area for future research.

In summary, small non-coding RNAs in spermatozoa constitute a largely unexplored area of epigenetic transgenerational inheritance. External environmental exposures and lifestyle factors can rapidly reprogram the mature spermatozoa RNA profiles by either interfering with intracellular mechanisms during spermatogenesis or through the loading of the RNAs from supporting tissues. As such, small non-coding RNAs can reprogram the developmental trajectories and physiology of the embryo, affect homeostatic mechanisms in adult offspring, and significantly contribute to the developmental origins of health and disease.

## Figures and Tables

**Table 2:** Overview of the sncRNAs found in mouse spermatozoa, their variable levels after environmental interventions, and their transgenerational effect.

Study	Intervention	Affected Molecule(s) in Sperm	Effect on the Offspring
Short et al. [25]	Exercise	Upregulated: 5′-tRFs and 5′-tRNA halves, miR-133. Downregulated: miR-190b, miR-19b	Reduced fear and anxiety
Guo et al. [30]	Aging	Upregulated: 69 tRFs, Downregulated: 35 tRFs	Higher anxiety and differential expression of signaling and developmental genes in brain and pre-implantation embryo
Chan et al. [20]	Corticosterone exposure or Chronic stress	Up-/Downregulated: 10 s of miRNAs	Increased body weight and stress responses as adults, deregulated expression of synaptic and immune response genes as embryos
Tomar et al. [27]	High-fat diet	Mitochondrial tRFs	Upregulation of oxidative phosphorylation in embryos
Sharma et al. [24]	Low-protein diet	5′-tRFs and 5′-tRNA halves from GlyGCC	MERVL targets in embryo
